# Prospective audit and feedback implementation by a multidisciplinary antimicrobial stewardship team shortens the time to de-escalation of anti-MRSA agents

**DOI:** 10.1371/journal.pone.0271812

**Published:** 2022-07-29

**Authors:** Ryo Yamaguchi, Takehito Yamamoto, Koh Okamoto, Keita Tatsuno, Mahoko Ikeda, Takehiro Tanaka, Yoshitaka Wakabayashi, Tomoaki Sato, Shu Okugawa, Kyoji Moriya, Hiroshi Suzuki

**Affiliations:** 1 Department of Pharmacy, The University of Tokyo Hospital, Tokyo, Japan; 2 Department of Infection Control and Prevention, The University of Tokyo Hospital, Tokyo, Japan; 3 The Education Center for Clinical Pharmacy, Graduate School of Pharmaceutical Sciences, The University of Tokyo, Tokyo, Japan; 4 Department of Infectious Diseases, The University of Tokyo Hospital, Tokyo, Japan; Azienda Ospedaliera Universitaria di Perugia, ITALY

## Abstract

Prospective audit and feedback (PAF) is considered an effective procedure for appropriate antibiotic use. However, its effect on the time to de-escalation is unclear. We aimed to evaluate the effect of daily PAF implementation, focusing on the time to de-escalation of anti-methicillin‐resistant *Staphylococcus aureus* (MRSA) agents as an outcome measure. To this end, a single-center, retrospective, quasi-experimental study including patients treated with intravenous anti-MRSA agents during pre-PAF (April 1, 2014 to March 31, 2015) and post-PAF (April 1, 2015 to March 31, 2016) periods was conducted. The time to de-escalation was estimated using the Kaplan–Meier method, and Cox proportional hazard analysis was performed to assess the effect of daily PAF implementation on the time to de-escalation. Interrupted time series analysis was used to evaluate the relationship between daily PAF implementation and anti-MRSA agent utilization data converted to defined daily dose (DDD) and days of therapy (DOT) per 1,000 patient days. The median time to de-escalation was significantly shorter in the post-PAF period than in the pre-PAF period (6 days *vs*. 7 days, *P* < 0.001). According to multivariate analysis, PAF implementation was independently associated with a shorter time to de-escalation (hazard ratio [HR], 1.18; 95% confidence interval [CI], 1.02 to 1.35). There were no significant differences in hospital mortality, 30-day mortality, and length of stay between the two periods. Interrupted time series analysis showed significant reductions in the trends of DDD (trend change, –0.65; 95% CI, –1.20 to –0.11) and DOT (trend change, –0.74; 95% CI, –1.33 to –0.15) between the pre-PAF and post-PAF periods. Daily PAF implementation for patients treated with intravenous anti-MRSA agents led to a shorter time to de-escalation and lower consumption of anti-MRSA agents without worsening the clinically important outcomes.

## Introduction

Antimicrobial resistance (AMR), along with the limited number of new antimicrobials in the drug-development pipeline, has been increasingly recognized as one of the greatest global public health threats [1]. In response to this circumstance, the World Health Assembly endorsed a global action plan to tackle AMR in May 2015 [2]. One of the main goals of the global action plan is to optimize the use of antimicrobial agents to prevent the emergence of AMR. In this regard, antimicrobial stewardship programs (ASPs) are considered one of the major strategies to achieve optimal selection of antimicrobial agents with respect to the appropriate indications, dosages, and durations that ensure safe and effective antimicrobial therapy with minimal risk of AMR development in bacteria, including methicillin‐resistant *Staphylococcus aureus* (MRSA) and multidrug-resistant *Pseudomonas aeruginosa* (MDRP) [3]. To date, the Infectious Diseases Society of America (IDSA) and Society for Healthcare Epidemiology of America (SHEA) have developed ASP guidelines [4, 5] in which pre-authorization and/or prospective audit and feedback (PAF) are strongly recommended as core components of an ASP.

Studies have shown that PAF implementation facilitates the appropriate selection of antibiotics for empirical therapy, reduces the consumption of broad-spectrum antibiotics [6], and decreases the incidence of infections by antibiotic-resistant organisms [7]. In addition, PAF implementation contributes to shortening the duration of antibiotic therapy [8, 9]. However, while an important goal of PAF implementation is to shorten the duration of treatment with empiric, broad-spectrum antibiotics through prompt de-escalation to optimize antibiotic therapy [4], few studies have evaluated the effect of PAF implementation on the time needed to de-escalate empirical antimicrobials (*e*.*g*., carbapenems and anti-MRSA agents).

In the University of Tokyo Hospital, a multidisciplinary antimicrobial stewardship team (AST) is assigned for daily PAF implementation for patients treated with intravenous anti-MRSA agents since April 1, 2015. We hypothesized that daily PAF implementation would facilitate appropriate antibiotic use and, consequently, shorten the time to de-escalation. To test this hypothesis, we evaluated the effect of daily PAF implementation on patients treated with intravenous anti-MRSA agents with a focus on the time to de-escalation as an outcome measure.

## Materials and methods

### Ethics statement

The institutional ethics review board of the Graduate School of Medicine and Faculty of Medicine, The University of Tokyo, approved the study protocol (approval No. 2529) and waived the need for obtaining written informed consent from each patient. This study was conducted in accordance with the ethical standards laid down in the 1964 Declaration of Helsinki and its later amendments.

### Setting and study population

We performed a single-center, retrospective, quasi-experimental study at the University of Tokyo Hospital, a tertiary care teaching hospital with 1,217 beds, to compare the appropriateness of the use of anti-MRSA agents during one-year periods before and after the initiation of daily PAF implementation (hereinafter referred to as the pre-PAF period and post-PAF period, respectively). As daily PAF implementation in our hospital began on April 1, 2015, the pre-PAF and post-PAF periods spanned from April 1, 2014 through March 31, 2015 and from April 1, 2015 through March 31, 2016, respectively.

All hospitalized patients treated with intravenous anti-MRSA agents between April 1, 2014 and March 31, 2016 were included in this study. The intravenous anti-MRSA agents used included vancomycin, teicoplanin, linezolid, daptomycin, and arbekacin. The study excluded patients who received anti-MRSA agents for surgical prophylaxis or whose medical chart was inaccessible to protect the confidentiality of medical information. In case a patient was re-admitted to the University of Tokyo Hospital and was re-administered intravenous anti-MRSA agents within the study period, we considered each treatment course separately.

### PAF

**Fig 1:**
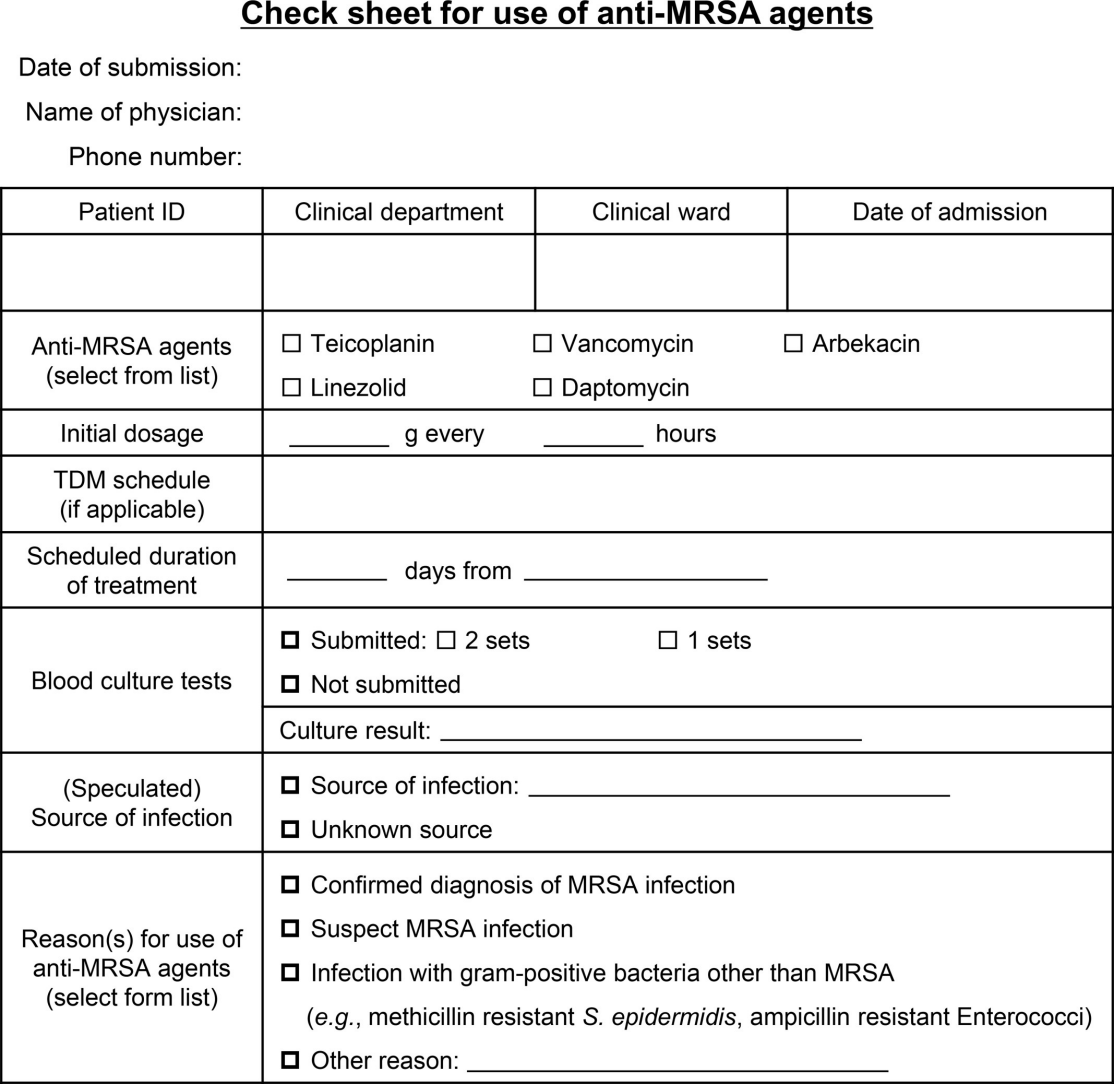
Check sheet for the use of anti-MRSA agents (English translation).

As part of the efforts to promote the appropriate use of antimicrobials in the University of Tokyo Hospital, since 2008, attending physicians have been requested to submit a check sheet (Fig 1) to the hospital infection control team through the electronic ordering system before the administration of intravenous anti-MRSA agents.

Before April 1, 2015, infection control team members conducted weekly reviews of patients treated with intravenous anti-MRSA agents. Simultaneously, one infectious disease specialist conducted daily reviews of patients with bacteremia. On April 1, 2015, the AST started daily PAF implementation for all patients who received intravenous anti-MRSA agents. The AST members comprised an infectious disease specialist, three clinical pharmacists, and one clinical microbiologist. Patients who newly started treatment with intravenous anti-MRSA agents were listed daily on weekdays. The AST members reviewed each patient on the day after the start of intravenous anti-MRSA agents and the day after the completion of the predetermined duration of anti-MRSA therapy. In each review, continued use of anti-MRSA agents was judged as potentially inappropriate in the following instances: 1) when microbiology test results showed no growth of MRSA, 2) in the absence of neutropenia or other immunosuppressive conditions, 3) beyond the recommended duration of therapy without any clinical reasons, and 4) in the absence of a contraindication to switch to oral antimicrobials. When the use of anti-MRSA agents was judged inappropriate, the AST members communicated this to the attending physicians on a face-to-face basis or by phone call. In addition, the AST recommended de-escalation when the antibiotic susceptibility of pathogens was reported.

### Definition of de-escalation

In this study, “de-escalation” was defined as ceasing anti-MRSA agents in the following two modes [10]: “discontinuation,” discontinuing unnecessary anti-MRSA agents (*e*.*g*., transition from concurrent therapy of vancomycin plus piperacillin/tazobactam to piperacillin/tazobactam monotherapy or changing vancomycin to cefazolin based on microbiology test results) and “oral switch,” switching to oral antibiotics (*e*.*g*., switching from vancomycin to oral trimethoprim-sulfamethoxazole).

### Data collection and outcome

Age, sex, intensive care unit (ICU) stay, MRSA carriage (defined as the recovery of MRSA from any body site, including nasal swab samples), and diagnosis of infections (determined by infectious disease specialists) were collected from the patients’ electronic medical charts. The diagnosis of infections was classified into the following categories: cardiovascular system infection (CSI), catheter-related blood stream infection (CRBSI), febrile neutropenia (FN), intraabdominal infection (IAI), urinary tract infection (UTI), respiratory tract infection (RTI), surgical site infection (SSI), skin and soft-tissue infection/bone and joint infection (SSTI/BJI), and other infections.

The primary outcome was time to de-escalation. Secondary outcomes were hospital-wide consumption of anti-MRSA agents, in-hospital mortality, 30-day mortality, length of stay in hospital (LOSH), duration of anti-MRSA agent therapy, reasons for discontinuation, incidence of adverse drug reactions (ADRs) documented by attending physicians, incidence of MRSA isolation, and the proportion of MRSA among all *S*. *aureus* isolates. Blood culture (BC) samples were incubated using the BacT/Alert system (bioMérieux, Lyon, France) and identified using the MicroScan WalkAway 96 plus System (Beckman Coulter, Brea, CA) and MALDI Biotyper (Bruker Daltonics, Billerica, MA).

The monthly consumption of anti-MRSA agents between April 2014 and March 2016 was converted into defined daily dose (DDD) using the Anatomical Therapeutic Chemical Classification System version 2017 developed by the WHO Collaborating Centre for Drug Statistics Methodology [11]. The DDD and days of therapy (DOT) were normalized to 1,000 patient days (PD). The incidence of MRSA isolation was calculated as the number of MRSA isolations per 1,000 PD.

### Statistical analyses

All statistical analyses were performed using SPSS version 24.0 (IBM, Armonk, NY). Categorical variables were compared using Pearson’s chi-square test or Fisher’s exact test, as appropriate. Continuous variables were compared using the unpaired Student’s *t*-test or Mann–Whitney U test. Residual analysis was conducted to assess the difference in the diagnosis of infections and the reasons for discontinuation between pre- and post-PAF periods. All statistical tests were two-tailed, and *P* < 0.05 was considered statistically significant.

The time to de-escalation was estimated using the Kaplan–Meier method and compared between groups using the generalized Wilcoxon test. Cox proportional hazard analysis was performed to identify variables affecting the time to de-escalation. The variables assessed were PAF implementation, age, sex, ICU stay, MRSA carriage, BC sample collection, and diagnosis of infections. In univariate analysis, variables with *P* < 0.1 were included for subsequent multivariate analysis. Additionally, to exclude the possible unintended effects of the data obtained immediately before/after the start of daily PAF, the time to de-escalation was estimated excluding those obtained within one month immediately before and after the start of daily PAF (*e*.*g*., April 2015-Feburary 2015 vs May 2015-March 2016).

Monthly DDD and DOT from the pre- and post-PAF periods were subjected to an interrupted time series analysis [12] to assess the longitudinal effect of PAF implementation on the DDD and DOT of anti-MRSA agents. A linear regression model was utilized to determine the magnitude of changes in the trends of DDD and DOT (*i*.*e*., slopes of DDD and DOT plotted against time) between the pre- and post-PAF periods and that of immediate changes in DDD and DOT at the transition point from the pre-PAF to the post-PAF period. Autoregressive integrated moving average models were used to evaluate the relationship between the consumption of anti-MRSA agents and PAF implementation.

## Results

### Patient characteristics and recommendations by the AST

**Fig 2:**
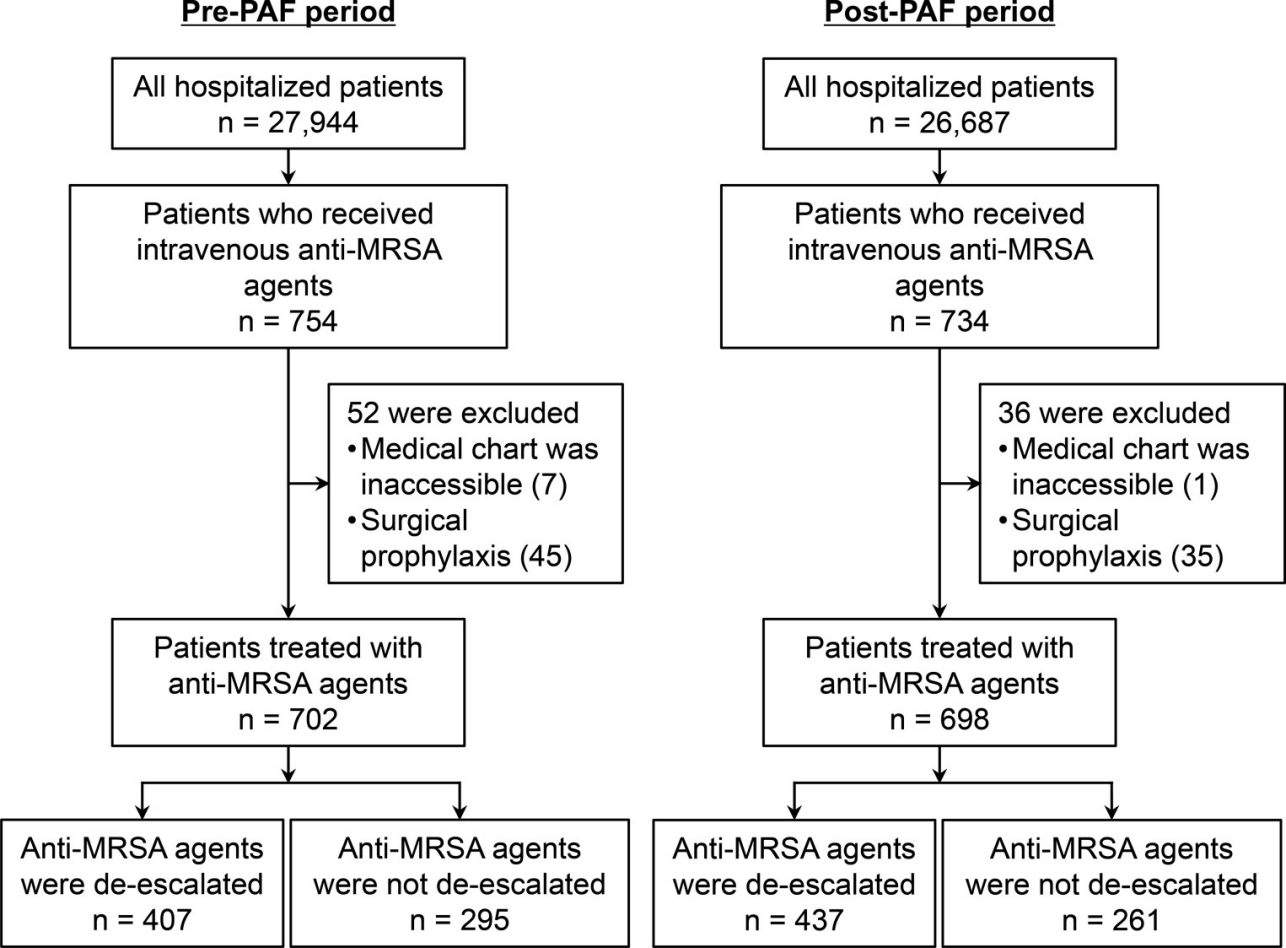
Flow chart of the selection of patients included in this study.

In total, 1,400 patients treated with anti-MRSA agents (n = 702 and 698 in the pre- and post-PAF periods, respectively) met the inclusion criteria (Fig 2). The most used anti-MRSA agents were vancomycin (63.0%) and teicoplanin (31.2%). Arbekacin was not used in both periods. The patient characteristics are summarized in Table 1. Significantly fewer patients stayed in the ICU during the pre-PAF period than during the post-PAF period (9.1% *vs*. 13.3%, *P* = 0.013). The proportion of MRSA carriers was significantly higher in the pre-PAF period than in the post-PAF period (23.1% *vs*. 17.6%, *P* = 0.011). BCs were obtained more frequently in the post-PAF period than in the pre-PAF period (91.8% *vs*. 86.5%, *P* = 0.001). In addition, there were significant differences in diagnosis of infections (*e*.*g*., FN, UTI, and other infections) between the pre- and post-PAF periods. Anti-MRSA agents were de-escalated to other antibiotics in 407 (58.0%) and 437 (62.6%) cases during the pre- and post-PAF periods, respectively (Table 1). Among the de-escalated cases, there were no significant differences in age, sex, proportion of ICU stay, and proportion of MRSA carriers between the pre- and post-PAF periods. In contrast, diagnosis of infections differed significantly between the two periods (*e*.*g*., FN and other infections).

**Table 1:** Characteristics of the patients included in this study.

Characteristics	All			De-escalation		
	pre-PAF (n = 702)	post-PAF (n = 698)	*P* value	pre-PAF (n = 407)	post-PAF (n = 437)	*P* value
Age [years], median (25^th^–75th percentile)	64 (43–74)	64 (42–73)	0.96	64 (44–73)	65 (43–73)	0.73
Male sex, n [%]	451 (64.2)	442 (63.3)	0.72	263 (64.6)	258 (59.0)	0.096
ICU stay, n [%]	64 (9.1)	93 (13.3)	**0.013**	42 (10.3)	58 (13.3)	0.19
MRSA carrier, n [%]	162 (23.1)	123 (17.6)	**0.011**	66 (16.2)	80 (18.3)	0.42
BC collected^a^, n [%]	607 (86.5)	641 (91.8)	**0.001**	362 (88.9)	405 (92.7)	**0.060**
De-escalation^b^, n [%]	407 (58.0)	437 (62.6)	0.077	-	-	-
Diagnosis of infections, n [%]			**<0.001**			**<0.001**
CSI	51 (7.3)	45 (6.4)	0.54	27 (6.6)	24 (5.5)	0.49
CRBSI	178 (25.4)	151 (21.6)	0.10	91 (22.4)	83 (19.0)	0.23
FN	121 (17.2)	88 (12.6)	**0.015**	82 (20.1)	64 (14.6)	**0.035**
IAI	108 (15.4)	93 (13.3)	0.27	63 (15.5)	65 (14.9)	0.81
UTI	21 (3.0)	38 (5.4)	**0.022**	16 (3.9)	24 (5.5)	0.29
RTI	59 (8.4)	64 (9.2)	0.61	33 (8.1)	41 (9.4)	0.51
SSI	69 (9.8)	56 (8.0)	0.24	34 (8.4)	32 (7.3)	0.58
SSTI/BJI	70 (10.0)	64 (9.2)	0.61	50 (12.3)	46 (10.5)	0.42
Other infections	25 (3.6)	99 (14.2)	**<0.001**	11 (2.7)	58 (13.3)	**<0.001**

*P* values that represent a significant difference are highlighted in bold.

^a^ Number of patients whose BC samples were collected before administration of anti-MRSA agents.

^b^ Number of patients whose anti-MRSA therapy was de-escalated.

The recommendations made by the AST are summarized in Table 2. The AST directly advised in 337 out of 698 cases (48.3%). Among them, the AST recommended de-escalation in 49 cases. Additionally, the AST recommended the primary team to shorten the duration of therapy in 105 cases, which might have encouraged de-escalation.

**Table 2:** Summary of recommendations by the AST.

Recommendation	Number of recommendations (%)
De-escalation	49 (14.5)
Shorten the duration of therapy	105 (31.2)
Discontinuation of antibiotic treatment because of negative diagnosis of infection	23 (6.8)
Discontinuation of anti-MRSA agents because of lack of clinical indications for their use	132 (39.2)
Other recommendations^a^	28 (8.3)
Total	337 (100)

^a^ Other recommendations included: consideration of consultation with infectious disease specialists, 7 cases; optimization of dosage, 11 cases; consideration of escalation, 4 cases; removal of catheters, 6 cases.

### Clinical outcomes of PAF implementation by the AST

**Fig 3:**
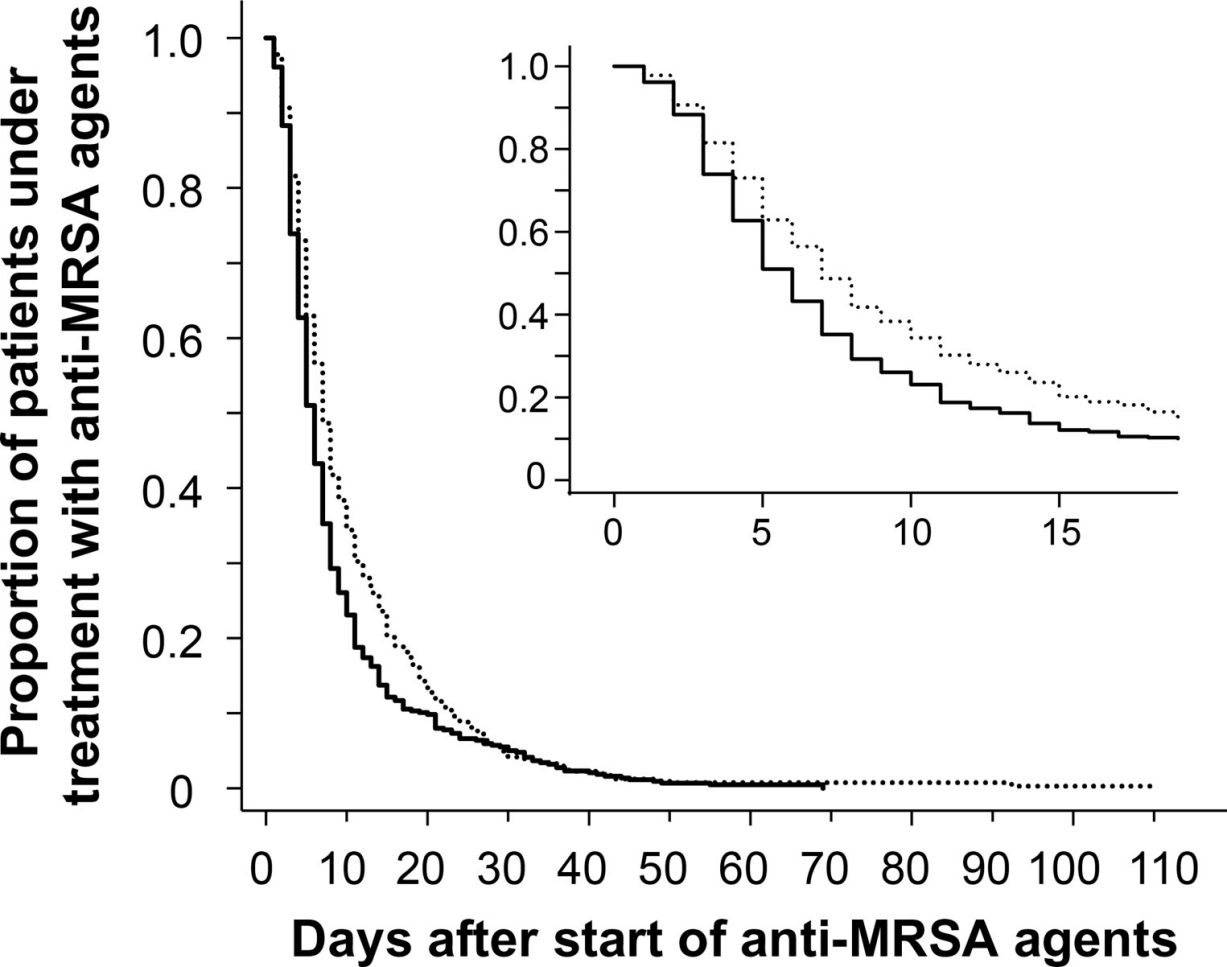
Kaplan–Meier curves showing the effect of PAF implementation on the time to de-escalation of anti-MRSA agents. The dotted and solid lines indicate the pre-PAF (n = 407) and post-PAF (n = 437) periods, respectively. The time to de-escalation was significantly shorter in the post-PAF period than in the pre-PAF period (median 6 days *vs*. 7 days, *P* < 0.001, generalized Wilcoxon test). The inset in the graph presents the same data on an enlarged horizontal axis.

**Fig 4:**
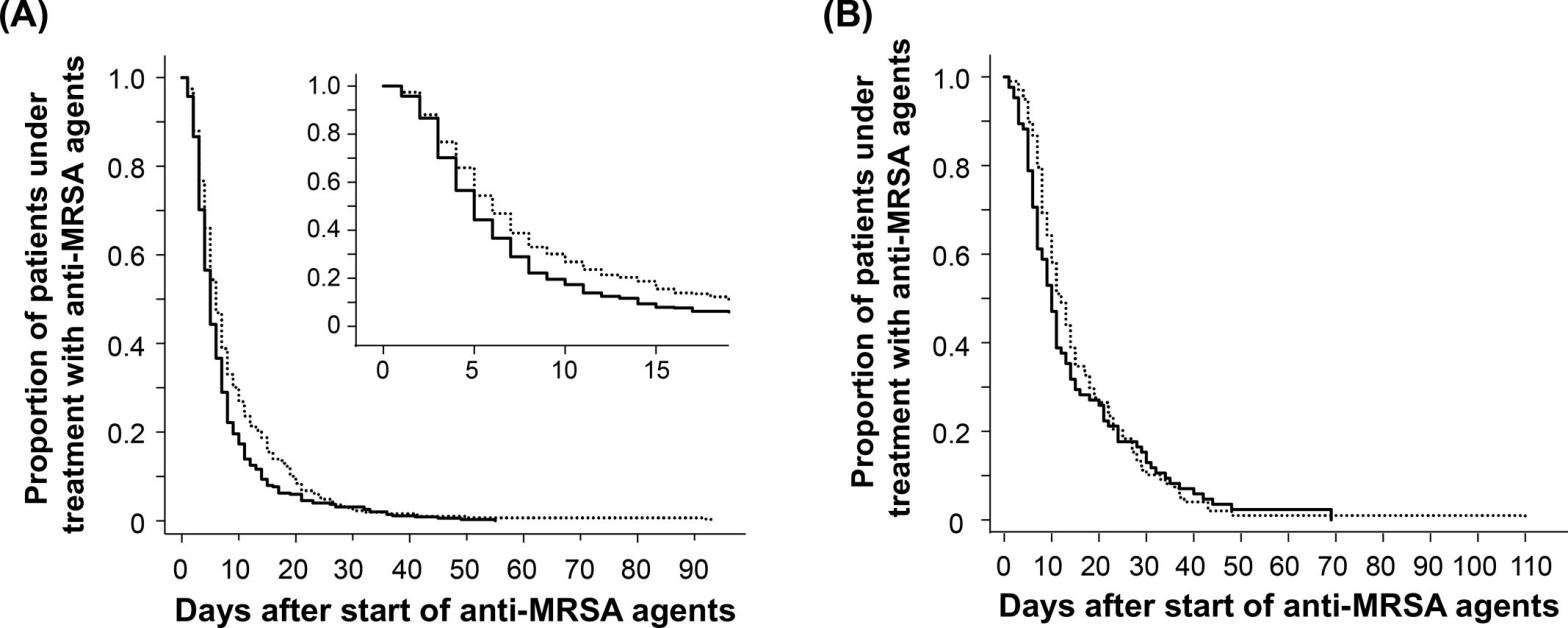
Subgroup analysis of the effect of PAF implementation on the time to de-escalation of anti-MRSA agents. The time to de-escalation in two subgroups was estimated using the Kaplan–Meier method. Panels A and B show Kaplan–Meier curves for the “discontinuation” and “oral switch” subgroups, respectively. The “discontinuation” subgroup included 309 and 352 cases from the pre- and post-PAF periods, respectively, and the “oral switch” subgroup included 98 and 85 cases from the pre- and post-PAF periods, respectively. Dotted and solid lines represent Kaplan–Meier curves for the pre- and post-PAF periods, respectively. PAF implementation significantly shortened the time to de-escalation (median 5 days *vs*. 6 days, *P* = 0.002, generalized Wilcoxon test) in the “discontinuation” subgroup. In contrast, PAF implementation had no significant effect on the time to de-escalation (median 10 days *vs*. 12 days, *P* = 0.082) in the “oral switch” subgroup. The inset in panel A shows the same data on an enlarged horizontal axis.

The median time to de-escalation was significantly shorter in the post-PAF period than in the pre-PAF period (6 days *vs*. 7 days, *P* < 0.001, generalized Wilcoxon test) (Fig 3). Analysis excluding one month immediately before and after the intervention also showed that the median time to de-escalation was statistically significantly shortened in the post-PAF period relative to the pre-PAF period (S1 Fig). Multivariate analysis revealed that PAF implementation (hazard ratio [HR], 1.18; 95% confidence interval [CI], 1.02 to 1.35), UTI (HR, 1.42; 95% CI, 1.00 to 2.00), and other infections (HR, 1.44; 95% CI, 1.08 to 1.91) were significantly associated with a shorter time to de-escalation (Table 3). In contrast, carriage of MRSA (HR, 0.70; 95% CI, 0.58 to 0.85), CSI (HR, 0.52; 95% CI, 0.38 to 0.72), FN (HR, 0.59; 95% CI, 0.47 to 0.74), IAI (HR, 0.76; 95% CI, 0.60 to 0.96), SSI (HR, 0.59; 95% CI, 0.44 to 0.79), and SSTI/BJI (HR, 0.65; 95% CI, 0.50 to 0.84) were significantly associated with a longer time to de-escalation (Table 3). Interestingly, the changes in the time to de-escalation after PAF implementation were different between the two modes of de-escalation. In the “discontinuation” cases, the time to de-escalation was significantly shorter in the post-PAF period than in the pre-PAF period (5 days *vs*. 6 days, *P* = 0.002) (Fig 4A). In contrast, in the “oral switch” cases, the time to de-escalation was not significantly different between the two periods; however, there was a trend towards shorter time to de-escalation post PAF implementation (10 days *vs*. 12 days, *P* = 0.082) (Fig 4B).

**Table 3:** Results of multivariate analysis of the time to de-escalation.

Variable	No. in pre-PAF (n = 407)	No. in post-PAF (n = 437)	Univariate analysis		Multivariate analysis	
			HR (95%CI)	*P* value	HR (95%CI)	*P* value
PAF, n [%]	0 (0)	437 (100)	1.24 (1.08–1.42)	0.002	1.18 (1.02–1.35)	**0.024**
Age ≥ 65 years, n [%]	190 (46.7)	219 (50.1)	1.04 (0.91–1.20)	0.54	-	-
Sex (male), n [%]	263 (64.6)	258 (59.0)	1.02 (0.89–1.17)	0.78	-	-
ICU stay, n [%]	42 (10.3)	58 (13.3)	0.91 (0.74–1.12)	0.36	-	-
MRSA carrier, n [%]	66 (16.2)	80 (18.3)	0.71 (0.59–0.85)	<0.001	0.70 (0.58–0.85)	**<0.001**
BC collected^a^, n [%]	362 (88.9)	405 (92.7)	1.36 (1.08–1.73)	0.010	1.20 (0.94–1.53)	0.14
**Diagnosis of infections, n [%]**						
CSI	27 (6.6)	24 (5.5)	-	**<0.001**	0.52 (0.38–0.72)	**<0.001**
CRBSI	91 (22.4)	83 (19.0)	-		reference	-
FN	82 (20.1)	64 (14.6)	-		0.59 (0.47–0.74)	**<0.001**
IAI	63 (15.5)	65 (14.9)	-		0.76 (0.60–0.96)	**0.021**
UTI	16 (3.9)	24 (5.5)	-		1.42 (1.00–2.00)	**0.049**
RTI	33 (8.1)	41 (9.4)	-		0.86 (0.65–1.13)	0.27
SSI	34 (8.4)	32 (7.3)	-		0.59 (0.44–0.79)	**<0.001**
SSTI/BJI	50 (12.3)	46 (10.5)	-		0.65 (0.50–0.84)	**<0.001**
Other infections	11 (2.7)	58 (13.3)	-		1.44 (1.08–1.91)	**0.013**

*P* values that represent a significant difference are highlighted in bold.

^a^ BC samples collected before the administration of anti-MRSA agents.

The effects of PAF implementation on secondary outcomes are summarized in Table 4. When all cases were analyzed, the duration of treatment with intravenous anti-MRSA agents was significantly shorter (7 days *vs*. 8 days, *P* < 0.001) and the incidence of ADRs was significantly lower (7.4% *vs*. 12.5%, *P* = 0.002) in the post-PAF period than in the pre-PAF period. There were no significant differences in in-hospital mortality, 30-day mortality, and LOSH. Analysis of the de-escalation cases revealed that the duration of treatment with intravenous anti-MRSA agents was significantly shorter in the post-PAF period than in the pre-PAF period (6 days *vs*. 7 days, *P* < 0.001). However, the other clinical outcomes (*i*.*e*., in-hospital mortality, 30-day mortality, and LOSH) were similar between the two periods.

**Table 4:** Effects of PAF implementation on secondary clinical outcomes.

	All cases			De-escalated cases		
	Pre-PAF (n = 702)	Post-PAF (n = 698)	*P* value	Pre-PAF (n = 407)	Post-PAF (n = 437)	*P* value
In-hospital mortality, n [%]	112 (16.0)	105 (15.0)	0.64	55 (13.5)	57 (13.0)	0.84
30-day mortality^a^, n [%]	53 (7.5)	57 (8.2)	0.67	14 (3.4)	26 (5.9)	0.086
LOSH [day], median (25^th^–75th percentile)	62 (37–106)	56 (32–102)	0.11	62 (37–107)	58 (34–98)	0.25
DOT [day], median (25^th^–75th percentile)	8 (5–14)	7 (4–11)	**<0.001**	7 (4–14)^d^	6 (3–10)^d^	**<0.001** ^**c**^
Reason of discontinuation, n [%]			0.12			-
De-escalation	407 (58.0)	437 (62.6)	0.077	407 (100)	437 (100)	-
Completion of treatment	168 (23.9)	153 (21.9)	0.37	-	-	-
Switch to other anti-MRSA agents	75 (10.7)	52 (7.4)	**0.035**	-	-	-
Death	29 (4.1)	23 (3.3)	0.41	-	-	-
Transfer to other hospital	17 (2.4)	19 (2.7)	0.72	-	-	-
Other reasons^b^	6 (0.9)	14 (2.0)	0.076	-	-	-
ADRs^c^, n [%]	88 (12.5)	52 (7.4)	**0.002**	29 (7.1)	19 (4.3)	0.082

*P* values that represent a significant difference are highlighted in bold.

^a^ 30-day mortality from the start of treatment with anti-MRSA agents.

^b^ Other reasons included: cases not actually infected, 4 and 8 cases in the pre- and post-PAF periods, respectively; cessation of aggressive treatment, 2 and 6 cases in the pre- and post-PAF periods, respectively.

^c^ Cases documented by attending physicians were counted.

^d^ These data are also shown in Fig 3.

### Consumption of anti-MRSA agents and drug-resistance

**Fig 5:**
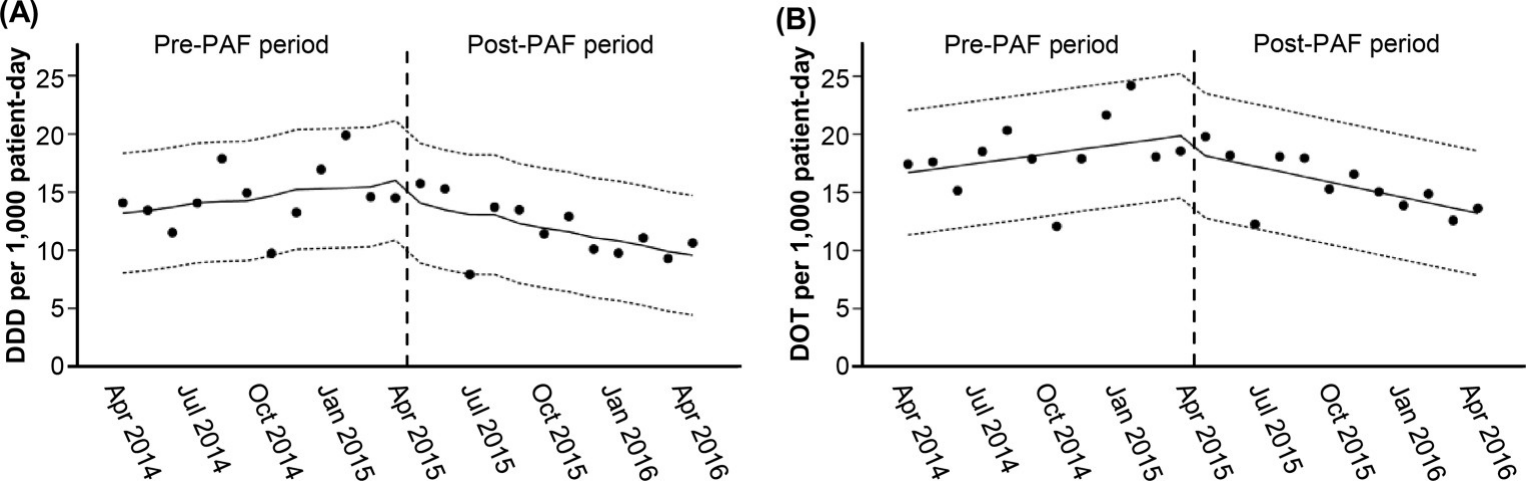
Effect of PAF implementation on the utilization of anti-MRSA agents. Time series of monthly DDD (panel A) and DOT (panel B) per 1,000 PD for anti-MRSA agents between April 2014 and March 2016 are plotted against time. Vertical dashed lines in each panel indicate the initiation of daily PAF implementation. Filled circles indicate the individual monthly DDD and DOT. Solid lines indicate the estimated trends during the study period according to interrupted time-series analysis, and the dotted lines indicate the upper and lower limits of the 95% confidence interval. Daily PAF implementation provoked a significant reduction in trends in both DDD (–0.65, 95% CI, –1.20 to –0.11, *P* = 0.029) and DOT (–0.74, 95% CI, –1.33 to –0.15, *P* = 0.024) of anti-MRSA agents.

The monthly DDD and DOT (normalized to 1,000 PD) of anti-MRSA agents decreased from 14.6 ± 2.7/1,000 PD to 11.8 ± 2.4/1,000 PD (mean ± SD, *P* = 0.015) and 18.3 ± 3.0/1,000 PD to 15.7 ± 2.4/1,000 PD (mean ± SD, *P* = 0.029), respectively. Interrupted time series analysis showed a significant reduction in the trends of DDD (trend change, –0.65; 95% CI, –1.20 to –0.11) and DOT (trend change, –0.74; 95% CI, –1.33 to –0.15) between the pre- and post-PAF periods. However, no significant difference was observed with respect to immediate changes in both DDD (6.26/1,000 PD; 95% CI, –1.51 to 14.03/1,000 PD) and DOT (7.58/1,000 PD; 95% CI, –0.88 to 16.03/1,000 PD) (Fig 5).

The incidence of MRSA isolation and proportion of MRSA in all *S*. *aureus* isolates were similar between the two periods (0.24/1,000 PD *vs*. 0.29/1,000 PD, *P* = 0.20 and 20% *vs*. 21%, *P* = 0.51, respectively).

## Discussion

This single-center, retrospective, quasi-experimental study revealed that daily PAF implementation for patients treated with anti-MRSA agents statistically significantly shortened the time to de-escalation and moreover reduced the consumption of anti-MRSA agents. To the best of our knowledge, this is the first study to evaluate the effect of daily PAF implementation on the time to de-escalation of anti-MRSA agents and to provide evidence of the benefit of daily PAF implementation by a multidisciplinary AST.

In our cohort, the time to de-escalation was shortened from 7 days to 6 days after daily PAF implementation (Fig 3). Similar results were obtained even after excluding one month immediately before/after the implementation from the analysis (S1 Fig). It is generally recognized that the best timing to consider de-escalation is 3–5 days after the initiation of empirical therapy because antibiotic susceptibility becomes available around this time. Indeed, Liu *et al* reported that the initial empirical antibiotic regimens are de-escalated to the definitive regimens within 4 days in most patients [13]. The difference in the time to de-escalation between our cohort and those in previous reports [13] may be attributable to the following two reasons. First, there was a difference in the incubation time of BC samples before finalization of the results. In our hospital, BC test results are generally finalized after 7 days if no growth is observed, which may have resulted in intravenous anti-MRSA agents being continued for 7 days in some patients. Several studies have shown that 5 days of incubation are sufficient for the routine detection of clinically significant organisms with the BacT/Alert system [14, 15]. Had the final culture result been reported 5 days after BC sample collection, the time to de-escalation may have been further shortened. Thus, speeding up bacteriological test results, regardless of whether the result is positive or negative, reduces the number of days of broad-spectrum antimicrobial use. AST interventions based on the results of matrix-assisted laser desorption ionization-time of flight mass spectrometry (MALDI-TOF MS) and multiplexed polymerase chain reaction (PCR) assays conducted by clinical microbiological technologists can lead to more timely antimicrobial therapy and shorter hospital stays [16, 17]. Currently, we are attempting to further reduce unnecessary anti-MRSA agents and promote early de-escalation using rapid diagnostic tests, including MALDI-TOF MS and multiplex PCR. The second reason is the difference in the definition of “de-escalation.” In the present study, “oral switch” was included in the definition of “de-escalation” in addition to “discontinuation.” Typically, switching intravenous anti-MRSA agents to oral antibiotics is considered after patients are treated with intravenous agents for a sufficient period [18]. As a result, the time to de-escalation tends to be longer when “oral switch” is included in the definition of “de-escalation.” Accordingly, in the subgroup analysis, where we separated cases involving “oral switch” from the “de-escalation” cases, the median time to de-escalation was significantly shorter in the post-PAF period (Fig 4).

Multivariate analysis revealed that not only daily PAF implementation but also the history of MRSA infection/colonization and diagnosis of infection were independent factors affecting the time to de-escalation (Table 3). A previous retrospective study showed that the colonization of multidrug-resistant organisms was associated with delayed de-escalation [19], which is concordant with our results. In our cohort, attending physicians tended to continue anti-MRSA agents until the BC results were finalized (7 days) in patients with a history of MRSA infection/colonization, irrespective of the results. Consequently, the time to de-escalation tended to be prolonged in these patients. Regarding the diagnosis of infections, FN, CSI, IAI, SSI, and SSTI/BJI significantly lengthened the time to de-escalation, whereas UTI and other infections significantly shortened the time to de-escalation. Several studies have reported the relationships between types of infection and the time to de-escalation. De Waele *et al*. reported that IAI was one of the reasons for a prolonged time to de-escalation [19], whereas Liu *et al*. reported that the proportion of patients whose antibiotic regimen was de-escalated within 72 hours was significantly higher for UTI than for SSTI [13]. The results of these previous studies are consistent with our findings in this study. To the best of our knowledge, few studies have reported the associations between FN, CSI, and SSI and the time to de-escalation. In patients with FN, attending physicians consider de-escalation based on the neutrophil count and the general condition of the patient rather than microbiology test results alone [20]. Therefore, the time to de-escalation is expected to be longer in patients with FN than in other patients.

Daily PAF implementation did not significantly affect clinically important outcomes, such as in-hospital mortality, 30-day mortality, and LOSH, in our cohort. These results are plausible because “de-escalation” is the action to discontinue antibiotics with limited clinical necessity; therefore, de-escalation seems to have a minimal effect on the clinical outcomes. A meta-analysis showed that the mortality associated with most infections was similar between de-escalation and non-de-escalation groups [21]. Further, in a systematic review of AST activities, only one study out of 10 observed a significant decrease in mortality upon PAF implementation by an AST, and no study demonstrated a significant decrease in the LOSH [22]. The findings in these previous reports are concordant with ours. In our cohort, the incidence of ADRs (kidney injury, drug eruption, and drug fever) significantly decreased after daily PAF implementation. Although few studies have investigated the correlation between the duration of therapy with anti-MRSA agents and the risk of ADRs, several studies have suggested an association between longer duration of therapy with anti-MRSA agents and higher risk of ADRs [23, 24]. Thus, it is possible that the shortening of the duration of therapy with anti-MRSA agents (8 days *vs*. 7 days, *P* < 0.001, Table 4) contributed to the reduced incidence of ADRs.

This study had some limitations. First, it was a retrospective quasi-experimental study. Therefore, although we included well-known confounders to the highest possible extent, there may have been potential unmeasured confounders. Second, this study was conducted at a single university hospital in Japan; thus, our findings may not be directly generalizable to other settings. Third, we did not include severity scores, including those of the sequential organ failure assessment and acute physiology and chronic health evaluation II. However, we included ICU stay as a surrogate indicator of severity, and it was not associated with the time to de-escalation. Additionally, we did not include comorbidities or concomitant medications, which might have affected the time to de-escalation. We cannot determine whether these factors may influence the results. Fourth, the incidence of ADRs may not reflect the true incidence, because ADRs were identified based on the documentation of the treating physicians. Further analysis is needed to investigate whether PAF implementation contributes to a reduction in the incidence of ADRs.

In conclusion, daily PAF implementation for patients treated with intravenous anti-MRSA agents significantly shortens the time to de-escalation and decreases the consumption of anti-MRSA agents, without affecting clinically important outcomes. Our findings provide evidence of the usefulness of PAF implementation by a multidisciplinary AST.

## Supporting information

S1 Fig Kaplan–Meier curves showing the effect of PAF implementation on time to de-escalation of anti-MRSA agents, excluding one month immediately before and after implementation. The dotted and solid lines indicate the pre-PAF (April 2015-Feburary 2015, n = 376) and post-PAF (May 2015- March 2016, n = 403) periods, respectively. The time to de-escalation was significantly shorter in the post-PAF period relative to the pre-PAF period (median 5 days vs. 7 days, P < 0.001, generalized Wilcoxon test). The inset in the graph presents the same data on an enlarged horizontal axis.
(TIF)
