# Supplementary figures and images for: Prospective audit and feedback implementation by a multidisciplinary antimicrobial stewardship team shortens the time to de-escalation of anti-MRSA agents
Source: PLoS One. 2022 Jul 29;17(7):e0271812. doi: 10.1371/journal.pone.0271812 (PMC9337637; doi:10.1371/journal.pone.0271812)

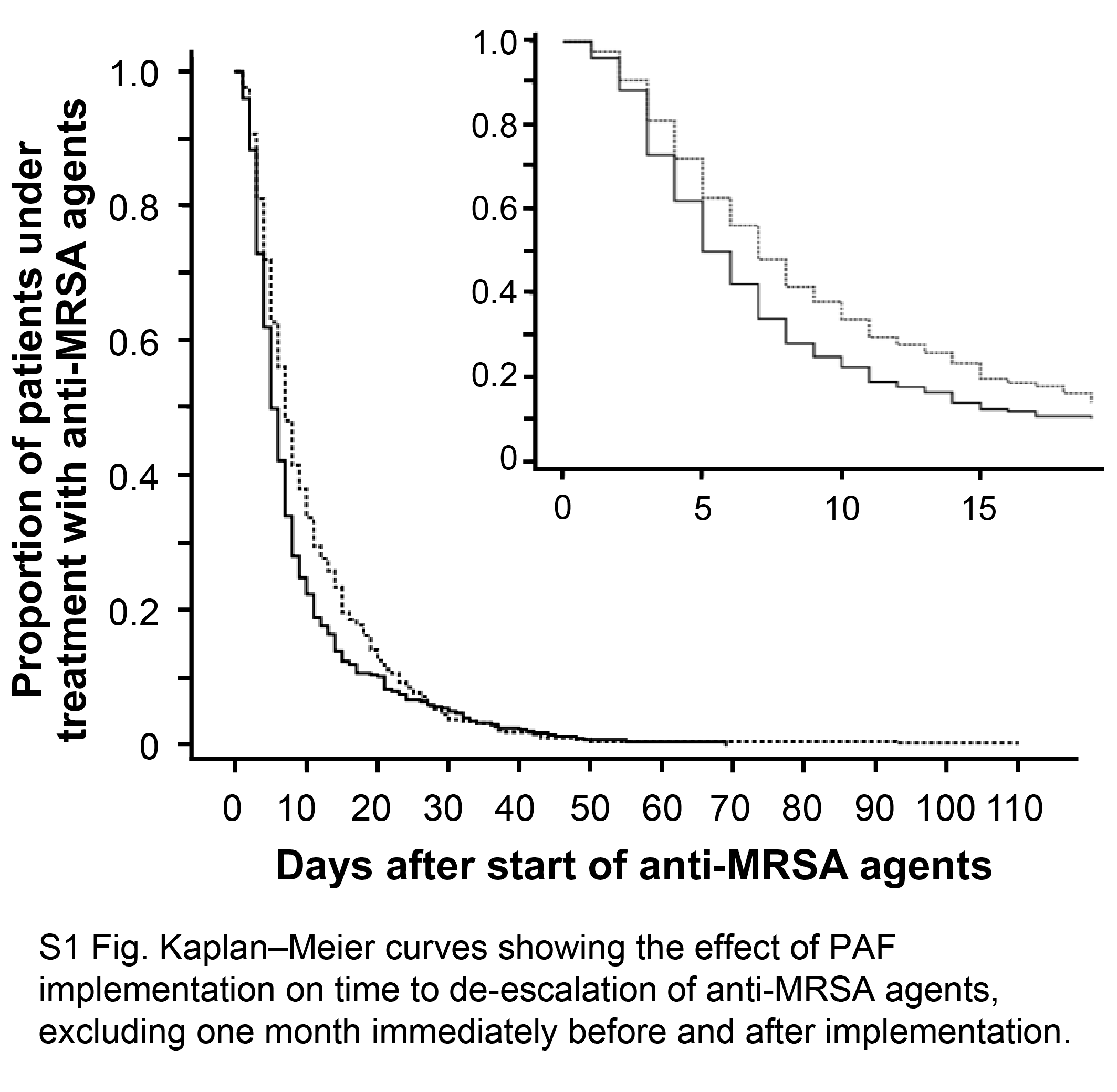

Supplement: S1 Fig — The dotted and solid lines indicate the pre-PAF (April 2015-Feburary 2015, n = 376) and post-PAF (May 2015- March 2016, n = 403) periods, respectively. The time to de-escalation was significantly shorter in the post-PAF period relative to the pre-PAF period (median 5 days vs. 7 days, P < 0.001, generalized Wilcoxon test). The inset in the graph presents the same data on an enlarged horizontal axis. (TIF) [file pone.0271812.s001.tif]
